# The Adventitia Resection in Treatment of Liver Hydatid Cyst: A Case Report of a 15-Year-Old Boy

**DOI:** 10.1155/2014/123149

**Published:** 2014-03-24

**Authors:** Zhenhua Ma, Wei Yang, Yingmin Yao, Qingguang Liu

**Affiliations:** Department of Hepatobiliary Surgery, First Affiliated Hospital, Medical School of Xi'an Jiaotong University, Xi'an 710061, China

## Abstract

Human hydatid disease is a significant health problem in endemic regions caused by the larval form of *Echinococcus granulosus*. In this paper, we report a case of liver hydatid cyst. The patient, a 15-year-old boy, presented with a history of intermittent upper abdominal pain of a few-month duration was referred to our hospital for investigation. Computed tomographic scan and laboratory test suggested a hydatid cyst in the right lobe of liver. The adventitia resection of hydatid cyst was smoothly performed as there was a less bloody virtual space between adventitia and outer membrane. Our diagnosis was made using an imaging approach and was confirmed during surgery. We proposed the adventitia resection of hydatid cyst could be safe and easy to perform with low risk of bleeding and bile leakage.

## 1. Introduction

Human hydatid disease, caused by the larval form of* Echinococcus granulosus*, is a significant health problem in endemic regions, especially in the Mediterranean countries, the Middle and Far East, Europe, Asia, South America, and Australia [1]. Incidental infestation leads to cyst formation in the liver. The most frequent complications of this severe disease include those related to the compression of adjacent organs or to perforation into the biliary system and pleural cavity or even to cyst infection [2]. Surgery is currently the primary treatment. It can be performed through laparoscopy or laparotomy. Radical surgical management of hepatic hydatid cysts includes ranges from aspiration of the parasite (or hydatid) to the excision of the cyst. The aim of the surgery is the total removal of the cysts without rupture since this can cause spillage of live scolices and anaphylaxis. The purpose of continuous improvement of the surgical method is to reduce the complication and to shorten the treatment period [3]. To achieve more effective evacuation of the cavity, we present the case of the adventitia resection.

## 2. Case Report

**Figure 1:**
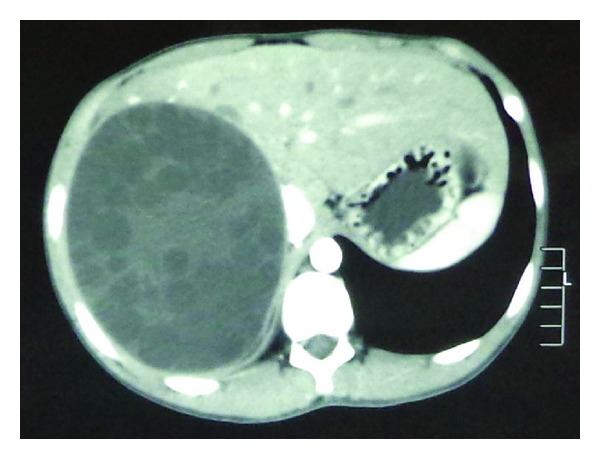
Computed tomographic scan of liver showing hydatid cyst.

**Figure 2:**
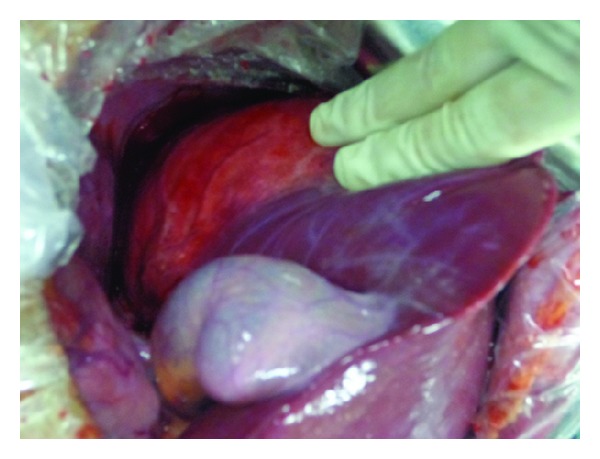
The liver hydatid cyst was in segments VII-VIII.

**Figure 3:**
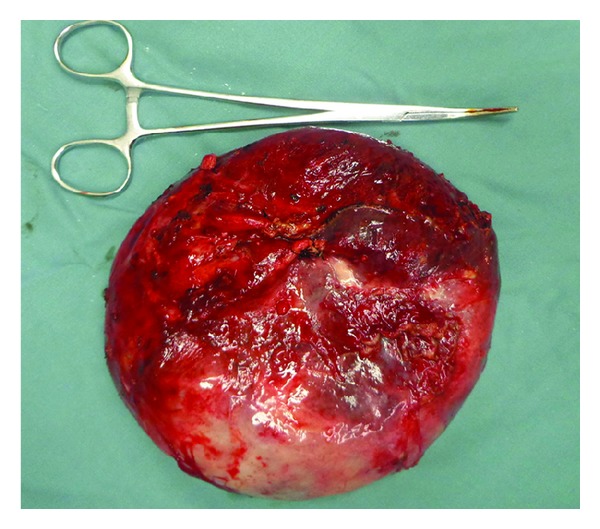
The hepatic hydatid cyst was stripped completely.

**Figure 4:**
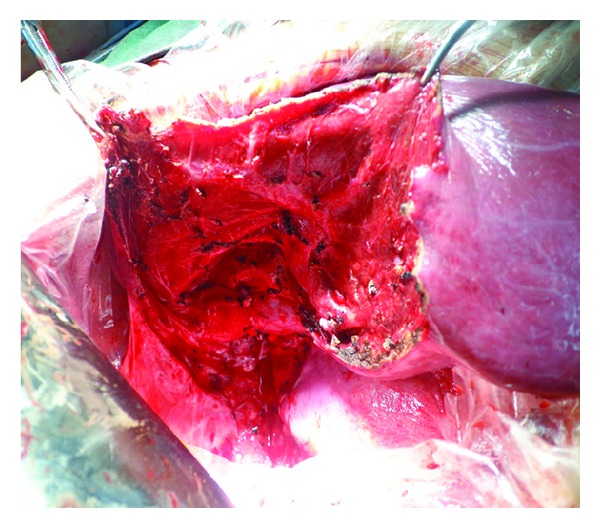
Operative view showing the intrahepatic Glisson system and hepatic venous system in right lobe.

The patient was a 15-year-old boy and presented with intermittent upper abdominal pain of a few-month duration. He described long-term rural life history and contacted with animals such as dog, cattle, and sheep. Upper abdominal CT indicated that the right lobe of liver had a large low-density lesion, suggesting liver hydatid cyst (Figure 1). Casoni test gave a positive reaction. Under general anesthesia, the adventitia resection of liver hydatid cyst was performed. The lesion was characterized by a 20 × 18 × 15 cm^3^ cystic mass in segments VII-VIII with fibrous membrane (Figure 2). A 12-French scale (Fr) percutaneous transhepatic drainage tube was inserted and the volume drained from the cyst was 60 mL to reduce cyst's pressure. Hepatic parenchymal dissection was performed in the edge of the lesion to divide the virtual space between adventitia and outer membrane. Then along this virtual space, we completely stripped the hepatic hydatid cyst and the lesion was completely free (Figures 3 and 4). Portal triad clamping was not performed at any stage of the operation. The intraoperative blood loss was 100 mL. The pathology report showed liver hydatid cyst tissue. Patients recovered smoothly and with no complications. The patient was followed up for 3 months and the evidence of recurrence was not found.

## 3. Discussion

Nowadays, hydatid disease is still a severe national problem in highly endemic countries and urgently needs epidemiologic prevention for its eradication [4]. Hydrated cyst is composed of three layers: adventitia, laminated membrane, and germinal layer. Adventitia is fibrous tissue about 1–3 mm thick induced by host's reaction against the hydatid cyst [3]. Following the hydatid cyst growing, apoptosis, degeneration or necrosis in the liver hepatocytes around the cyst occurred. It is observed in the periphery of the lesion that the intrahepatic Glisson system and hepatic venous system and their belonging fiber structure are extruded around adventitia, which resulted in continuous hyperplasia and fibrosis of these tissues, and eventually, outer membrane are formed. Wu et al. [5] suggested that there was a less bloody virtual space between adventitia and outer membrane. In the clinical practice, liver hydatid cyst can be integrally stripped along this virtual space.

The symptoms of hydatid disease are varied depending on which organs are affected. The most commonly affected organ is the liver in adults and lung in children. Surgery remains the primary method of treatment for hydatid disease [6]. Surgical procedures include partial cystectomy, cystectomy, pericystectomy, and hepatic resection using open or laparoscopic approach [7, 8]. In partial cystectomy, the cyst was unroofed and the residual cavity was managed with external tube drainage and the laminated membrane and germinal epithelium were partly removed. Thus, There is the possibility of planting hydatid scolex in peritoneal and residual cavities. That led to a high rate of recurrence [9, 10]. The rate of recurrence was significantly lower after radical surgical procedures (pericystectomy and hepatic resection). But pericystectomy and hepatic resection are described with a nonnegligible intraoperative risk for a benign disease including bile leakage and bleeding [7]. By the adventitia resection, we can completely remove the cyst and decrease the risk of recurrence postoperatively. The adventitia was completely removed in this operation without cyst fluid spills thus solving the recurrence caused by the residual of hydatid scolex in abdominal cavity.

## 4. Conclusion

The stripping process along the virtual space is smooth and can minimize the damage of the liver parenchyma to protect the intrahepatic pipeline system to avoid bleeding and bile leakage.
